# PI3K/AKT/mTOR Signaling-Mediated Neuropeptide VGF in the Hippocampus of Mice Is Involved in the Rapid Onset Antidepressant-Like Effects of GLYX-13

**DOI:** 10.1093/ijnp/pyu110

**Published:** 2015-03-04

**Authors:** Yang Lu, Chuang Wang, Zhancheng Xue, Chenli Li, Junfang Zhang, Xin Zhao, Aiming Liu, Qinwen Wang, Wenhua Zhou

**Affiliations:** Department of Pharmacology, and Provincial Key Laboratory of Pathophysiology in Ningbo University School of Medicine, Ningbo, Zhejiang, PR China.

**Keywords:** depression, GLYX-13, PI3K/AKT/mTOR signaling, VGF (nonacryonimic)

## Abstract

**Background:**

VGF (nonacryonimic) and phosphatidylinositol 3-kinase (PI3K)/AKT (also known as protein kinase B, PKB)/mammalian target of rapamycin (mTOR) signaling play pivotal roles in depression. However, whether phosphatidylinositol 3-kinase/AKT/mTOR signaling-mediated VGF participates in rapid-acting antidepressant-like actions of GLYX-13 is unclear.

**Methods:**

Herein, we evaluated the effects of acute treatment of GLYX-13 (0.5, 5, and 10mg/kg, i.p.) in the forced swim test. In addition, we assessed whether the acute treatment with GLYX-13 reverses the depressive-like behaviors induced by chronic unpredictable mild stress. Furthermore, we determined whether the *Vgf* knockdown in hippocampus of mice blocks the effects of GLYX-13. Moreover, we also demonstrated the effects of intra-hippocampus infusion of LY294002 (10 nmol/side), a specific phosphatidylinositol 3-kinase inhibitor prior to the treatment of GLYX-13 in the forced swim test. Lastly, whether alpha-amino-3-hydroxy-5-methyl-4-isoxazolepropionic acid (AMPA) receptor and mTOR activation involves in the antidepressant-like effects of GLYX-13 was examined.

**Results:**

Our results shown that GLYX-13 dose-dependently reversed the depressive-like behaviors in forced swim test. Additionally, GLYX-13 significantly reversed the downregulation of phosphorylation of AKT, mTOR, and eukaryotic elongation factor 2 as well as VGF induced by chronic unpredictable mild stress in hippocampus. Further, *Vgf* knockdown in hippocampus of mice significantly blocked the rapid-acting antidepressant-like effects and upregulation on phosphatidylinositol 3-kinase/AKT/mTOR/VGF signaling of GLYX-13. Moreover, intra-hippocampus infusion of LY294002 significantly abolished the antidepressant-like effects and upregulation on phosphatidylinositol 3-kinase/AKT/mTOR/VGF signaling of GLYX-13. Finally, antidepressant-like effects of GLYX-13 required AMPA receptor and mTOR activation, as evidenced by the ability of NBQX and rapamycin to block the effects of GLYX-13, respectively.

**Conclusions:**

Our results suggest that phosphatidylinositol 3-kinase/AKT/mTOR signaling-mediated VGF in hippocampus may be involved in the antidepressant-like effects of GLYX-13.

## Introduction

Depression is a serious public health problem and one of the most common psychiatric disorders, with a lifetime prevalence of 17% in the United States (Kessler et al., 2005). Notably, to date, all drugs approved as antidepressant medications are either inhibitors of monoamine transporters or monoamine oxidase (Berton and Nestler, 2006) and take 3 to 8 weeks to exert their effects (Wong and Licinio, 2004). Patients that emerge as treatment resistant, defined as failing 2 or more trials of medication, are more severely ill with comorbid anxiety disorders and are at increased risk of suicide for an extended period of time (Schosser et al., 2012). Therefore, there is a pressing medical need to develop rapidly acting therapeutics that are capable of immediately relieving the depressive symptomology.

Recently it has been demonstrated that the *N*-methyl-D-aspartic acid (NMDA) receptor (NMDAR) antagonist ketamine has rapid-acting antidepressant effects in patients that are treatment resistant (Mathew et al., 2012). Previous studies suggest that the rapid-acting antidepressant-like effects of ketamine are mediated by molecular alterations to the signaling pathway of phosphatidylinositol 3-kinase (PI3K)/AKT (also known as protein kinase B, PKB)/glycogen synthesis kinase 3 (GSK3)/mammalian target of rapamycin (mTOR)/brain derived neurotrophic factor (BDNF), which has been implicated in the adaptive response to stress, and are engaged in the development of mood-related disorders (Yang et al., 2013; Zhou et al., 2013; Abelaira et al., 2014; Park et al., 2014; Zhou et al., 2014). Autry and colleagues (2011) showed that ketamine and other NMDAR antagonists produce fast-acting behavioral antidepressant-like effects in mouse models and that these effects depend on the rapid synthesis of BDNF, which may play an important role on the antidepressant modulator neuropeptide VGF (nonacronymic) production (Alder et al., 2003; Malberg and Monteggia, 2008). These findings indicate that the regulation of protein synthesis by spontaneous neurotransmission may serve as a viable therapeutic target for the discovery of fast-acting antidepressants. Unfortunately, ketamine causes unacceptable dissociative side effects and is a substance of abuse (Burgdorf et al., 2013). Recent studies demonstrate that GLYX-13, an NMDAR glycine site functional partial agonist, produces rapid onset antidepressant-like effects without the psychotomimetic side effects of NMDAR antagonists (Burgdorf et al., 2013; Moskal et al., 2014). However, it remains to be revealed whether PI3K/AKT/mTOR also participates in the rapid-acting antidepressant-like effects of GLYX-13. Additionally, given that the VGF may act downstream of BDNF and exert rapid-acting antidepressant-like effects through TrkB receptor (Alder et al., 2003; Lin et al., 2014) and enhancing neurogenesis (Thakker-Varia et al., 2014) in hippocampus, many questions remain to be answered about the role of the VGF in antidepressant-like effects of GLYX-13.

## Materials and methods

### Animals

Experiments were conducted on young, healthy, male ICR mice (22–25g) born and reared in the animal facility of Ningbo University Medical School and Zhengzhou University, China. All animals were maintained at 22±2°C and 60% ± 5% relative humidity under a 12-h-light/-dark cycle (lights on at 7:00 am) with ad libitum access to food and water when the stressors were not applied. All stressors were applied to animals outside of their housing area in a separate procedure room. All animal experiments were performed according to the National Institutes of Health (NIH) Guide for the Care and Use of Laboratory Animals (NIH Publications No. 80-23, revised 1996) and were approved by the Institutional Animal Care and Use Committee of Ningbo University Medical School and Zhengzhou University.

### Drugs and Treatment

The drugs used included:GLYX-13 (Tocris Bioscience) and desipramine (Des) (Sigma, St. Louis, MO). Previous studies demonstrate that a low dose of dimethyl sulfoxide (DMSO) in saline vehicle did not make the changes in pharmacological effects and toxic effects of compounds (Wang et al., 2012; Li et al., 2011); both GLYX-13 and Des were dissolved in 0.9% saline containing 1% DMSO in our current work. These solutions, freshly prepared before administration, were given by i.p. in a volume of 10mL/kg body weight. LY294002, NBQX, and rapamycin were purchased from Tocris Bioscience (Tocris Bioscience), dissolved in artificial cerebrospinal fluid (ACSF), and administrated by intra-hippocampal (i.h.) infusion. The i.h. injection was performed by employing a “free hand” method under light ether anesthesia according to the procedure described previously (Zhang et al., 2013; Lin et al., 2014). Briefly, animals were anaesthetized with ketamine and xylazine (100 and 10mg/kg i.p., respectively) and placed in a stereotaxic frame in the flat-skull position. Stainless steel guide cannulae were implanted bilaterally into the hippocampal at AP −1.5mm from bregma, ML ± 1.2mm from the midline, and DV −2.0mm from dura. The guide cannulae were anchored to the skull with dental cement, and a stainless steel stylet was inserted to maintain patency for microinjection. The mice were allowed to recover for 7 days and were handled every other day to reduce the stress associated with handling at the time of testing. The drugs were then microinjected into the bilateral hippocampus of the mice. The injection cannula was left in place for another 60 seconds before being slowly withdrawn to avoid back flow.

### Chronic Unpredictable Mild Stress (CUMS)

This animal model of stress consists of chronic exposure to variable unpredictable stressors, none of which is sufficient alone to induce long-lasting effects. Briefly, CUMS consisted of exposure to a variety of unpredictable stressors (randomly), as shown in Table 1. The control animals were housed in a separate room and had no contact with the stressed groups. To prevent habituation and ensure the unpredictability of the stressors, all stressors were randomly scheduled during a 1-week period and repeated throughout the 3-week experiments.

**Table 1.** Schedule of Stressors Used in the 21 Days of CUMS Procedure

Stressor	Duration	Day
Food deprivation	24 h	Monday
Exposure to a foreign object	24 h	
Water deprivation	24 h	
Forced swimming at 12°C	6 min	Tuesday
Soiled cage	24 h	
Overnight illumination	Overnight	
Food deprivation	24 h	Wednesday
Cage tilt (4°C)	7 h	
Physical restraint	2 h	
Exposure to an empty bottle	1 h	Thursday
Cage tilt (45℃)	7 h	
Overnight illumination	Overnight	
Soiled cage	24 h	Friday
Forced swimming at 12℃	6 min	
Physical restraint	2 h	
Exposure to a foreign object	24 h	Saturday
Forced swimming at 12℃	6 min	
Cage tilt (45℃)	7 h	
Soiled cage	24 h	Sunday
Exposure to an empty bottle	1 h	
Overnight illumination	Overnight	

Abbreviation: CUMS, chronic unpredictable mild stress.

### Construction of *Vgf*-shRNA-Lentivirus

Small interfering RNAs targeting the mouse *Vgf* gene were designed by the Shanghai GeneChem, Co. Ltd, China. The optimal sequence of small interfering RNAs against mice VGF (5’-CCAATTCCAGGCTCGAATG-3’) was then cloned into the plasmid pGCL–GFP, which encodes an human immunodeficiency virus (HIV)-derived lentiviral vector containing a multiple cloning site for insertion of shRNA constructs to be driven by an upstream U6 promoter and a downstream cytomegalovirus promoter-GFP fluorescent protein (marker gene) cassette flanked by loxP sites. A negative control lentiviral vector containing *NS-*shRNA was constructed by a similar process (*NS-*lentivirus, 5’-TTCTCCGAACGTGTCACGT-3’). These modified plasmids were further cotransfected into HEK 293T cells with lentiviral packaging plasmids to generate an *Vgf-*shRNA-expressing lentivirus (*Vgf*-shRNA-lentivirus) or a control shRNA-expressing lentivirus. HEK293 cells were plated in 6-well plates (6×10^5^ cells/well) and cultured for 24 hours before the transduction of lentiviral vectors. After 2 days of infection, the medium was replaced and cells were further incubated until 48 hours as required.

### Experimental Treatments

**Figure 1.**
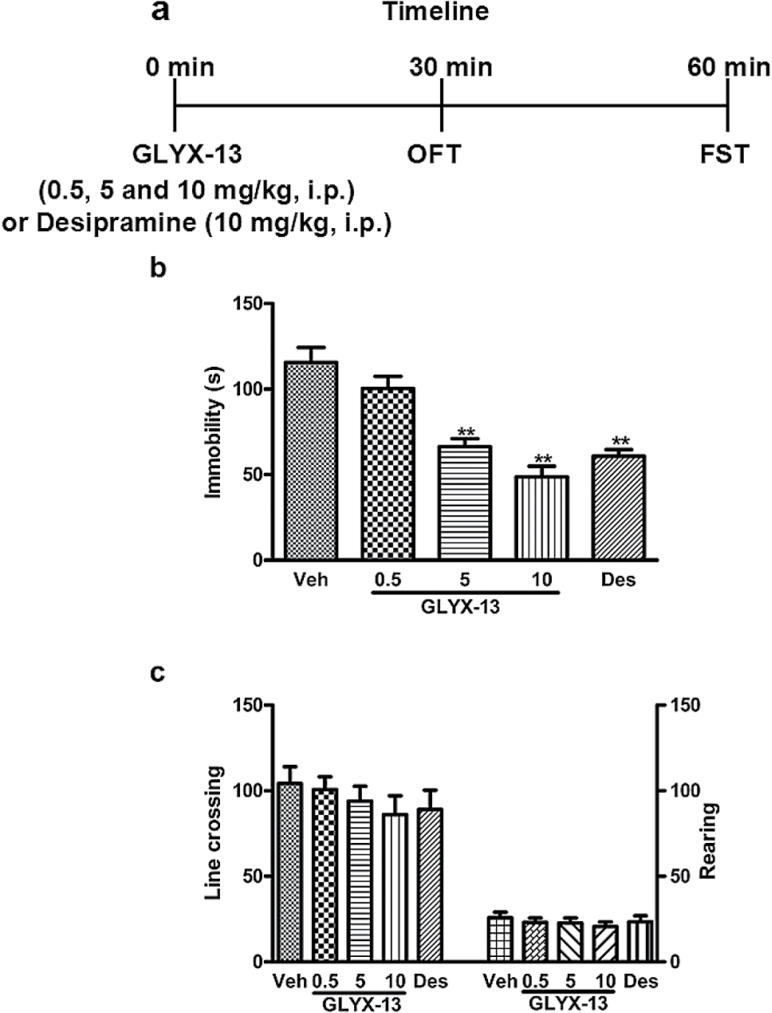
Antidepressant-like effect of acute GLYX-13 treatment in mice. (a) Protocol for experiments using the open field test (OFT) and forced swim test (FST). The OFT was conducted 30 minutes after a single injection of vehicle, GLYX-13 (0.5, 5, and 10mg/kg, i.p.), or Desipramine (Des) (10mg/kg, i.p.). The FST was performed 30 minutes after the OFT. (b) Acute GLYX-13 (5 and 10mg/kg, i.p.) treatment significantly decreased immobility time in the FST. (c) Acute GLYX-13 (0.5, 5, and 10mg/kg, i.p.) treatment had no effects on locomotor activity, reflected by the line crossing (left) and rearing (right) in mice. The data are expressed as mean±SEM (n=9 per group). **P<.01, compared with vehicle-treated group.

**Figure 2.**
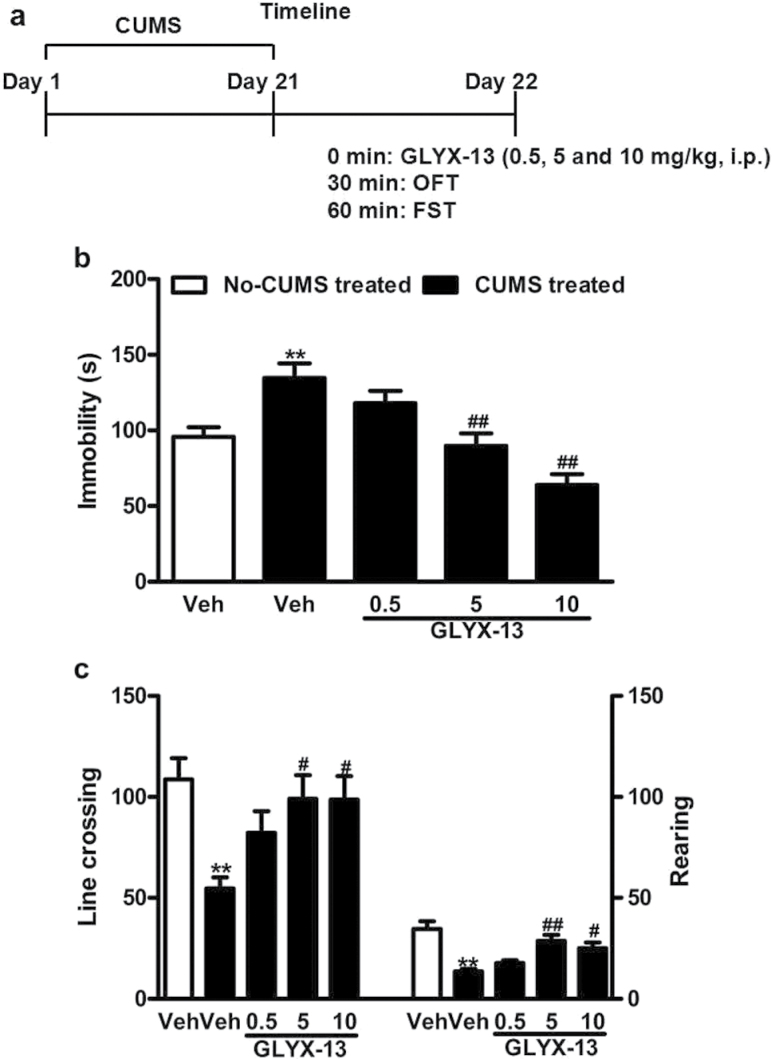
Effects of acute GLYX-13 administration on depressive-like behaviors induced by chronic unpredictable mild stress (CUMS) in mice. (a) Behavioral test procedure. Briefly, the mice were subjected to different stressors for 21 consecutive days. On day 22, the open field test (OFT) was conducted 30 minutes after a single injection of vehicle or GLYX-13 (0.5, 5, and 10mg/kg, i.p.). The forced swim test (FST) was performed 30 minutes after the OFT. (b) Acute GLYX-13 (5 and 10mg/kg, i.p.) treatment significantly reversed the depressive-like behaviors induced by CUMS in the FST. (c) GLYX-13 treatment (5 and 10mg/kg, i.p.) significantly reversed the decrease of line crossings (both f *P<.*01) and rearings (both *P<.*01) induced by CUMS. However, 3 doses of GLYX-13 had no significant effect on line crossings (all *P* > .05) and rearings (all *P* > .05) compared with each other. The data are expressed as mean±SEM (n=9 per group). **P<.01, compared with nonstress treated mice with vehicle-administrated group; ##*P<.*01, compared with stress treated mice with GLYX-13-administrated group.

Five sets of experiments were conducted. The first set was performed to assess the fast-acting antidepressant-like effects of acute GLYX-13 administration (Figure 1a). The second set of experiments was to investigate whether 21-day CUMS alters VGF and PI3K/AKT/mTOR signaling in the hippocampus of mice and to determine whether acute treatment of GLYX-13 (0.5, 5, and 10mg/kg, i.p.) reverses the depressive-like behaviors induced by CUMS in mice (Figure 2a). The third set was to demonstrate whether knock down of VGF in hippocampus by *Vgf*-shRNA-lentivirus blocks the antidepressant-like action of GLYX-13 (10mg/kg, i.p.) (Figure 4a). Lentiviral vectors containing *NS-*shRNA or *Vgf*-shRNA-lentivirus (5×10^7^ TU/μL, 1 μL/side) were infused at a rate of 0.2 μL/min with an infusion pump, and the cannulas were left in place for 60 additional seconds to avoid back flow. The fourth set of experiments was to investigate whether i.h. infusion of LY294002 (10 nmol/side), a specific PI3K inhibitor, 30 minutes prior to the administration of GLYX-13 (10mg/kg, i.p.) significantly blocks the antidepressant-like behaviors in the open field test (OFT) (Figure 6a). The fifth set of experiments was to investigate whether i.h. infusions of NBQX (2 μg/side), a AMPA receptor inhibitor (NBQX), or mTOR inhibitor (rapamycin) 30 minutes prior to the administration of GLYX-13 (10mg/kg, i.p.) blocks the antidepressant-like behaviors of GLYX-13 (Figure 8a, e).

### OFT

The OFT was conducted first to ensure that any changes in activity during the forced swim test (FST) were not due to nonspecific changes in motor activity. Briefly, mice were placed individually in a white Plexiglas box (50×50×39cm) with a bottom divided into 4 identical squares. Line crossings (4 paws placed into a new square) and rearings (with both front paws raised from the floor) were recorded for 5 minutes in a dimly lit room. After each test, the apparatus was cleaned with 5% ethanol to remove scent clues.

### FST

The FST was conducted in a sound-attenuated room eliminated by white light (40 lux) as described (Sarkisyan et al., 2010). Briefly, mice were placed individually in a clear plastic cylinder (height:25cm; diameter:10cm) containing 10cm of fresh water at 23±2°C for 6 minutes, and the duration of immobility was scored during the last 4 minutes. The total time during which the mouse made only small movements necessary to keep the head above water was considered the duration of immobility.

### Quantitative RT-PCR

The hippocampus tissues of 2mm in diameter around the injection site were punched out for quantitative RT-PCR (qRT-PCR). This was performed following the procedures described previously (Vahdati et al., 2014). Total RNAs were extracted from HEK293 cells (Figure 4b-c) or mice hippocampi (Figure 4 d-e) using High Pure RNA Tissue Kit according to the manufacturer’s instructions. qRT-PCR was performed to analyze transcript levels of VGF using EXPRESS One-Step SYBR GreenER SuperMix Kit for 1-step qRT-PCR according to the manufacturer’s instructions and a StepOne RT-PCR System (ABI). The following protocol was used:activation of reverse transcriptase and cDNA synthesis (50°C for 5 minutes), PCR activation (95°C for 2 minutes), 40 cycles of denaturation (95°C for 15 seconds), and annealing/extension (60°C for 1 minute). The primers used are as follows:VGF, forward 5′-GATGACGACGACGAAGAC-3’ and reverse 5′-CGATGATGCTGACCACAT-3′; β-actin, forward 5′-GGGA AATCGTGCGTGACATT-3′ and reverse 5′- GCGGCAGTGGCCATC TC-3′. The results are expressed as 2^-△*Ct*^ [△*Ct*=*Ct* (VGF) – *Ct* (β-actin)] and normalized by the level of β-actin.

### Immunoblotting

The hippocampus tissues of 2mm in diameter around the injection site were punched out for Western-blotting analysis. Brain tissues were sonicated in RIPA lysis buffer (Upstate, Temecula, CA) containing protease and phosphatase inhibitors (Pierce Biotechnology, Rockford, IL). Lysates were centrifuged at 16,000 × *g* for 30 minutes and total supernatant protein (80 μg gel lane) separated by SDS-PAGE and transferred to PVDF membranes (0.22 μm; Millipore, CA). Membranes were then incubated with rabbit anti-phospho-AKT-Ser473 (1:1000; Cell Signaling, Danvers, MA), rabbit anti-total-AKT (1:1000; Cell Signaling), rabbit anti-phospho-mTOR (1:1000; Millipore), rabbit anti-phospho-total-mTOR (1:1000; Abcam, Cambridge, MA), rabbit anti-phospho-eEF2 (1:800; Abcam), rabbit anti-VGF (1:500; Millipore), or anti-β-actin (1:1000; Chemicon) at 4°C overnight. The membranes were then incubated with Alexa Fluor 700-conjugated goat anti-rabbit antibody (1:10000; Invitrogen, Eugene, OR) for 60 minutes. Target bands were detected and quantified using a fluorescence scanner (Odyssey Infrared Imaging System, LI-COR Biotechnology, Lincoln, NE). All the lysate samples were analyzed at least in triplicate.

### Data Analysis

Data are expressed as the means ± SEM. Data were analyzed by 1-way analysis of variance (ANOVA) or 2-way ANOVA followed by Newman–Keuls posthoc test using GraphPad Prism software (Version 5.0, Prism software for PC, GraphPad). The criterion for significance was *P<*.05.

## Results

### GLYX-13 Administration Exerts Rapid-Acting Antidepressant-Like Effects in Mice

Using the FST in mice, we first assessed the antidepressant-like effects of acute GLYX-13 administration. The mice received an i.p. injection of GLYX-13 (0.5, 5, and 10mg/kg) or Des (10mg/kg) 30 minutes before the OFT and then immediately followed by the FST 30 minutes later (Figure 1a). One-way ANOVA revealed that GLYX-13 significantly decreased immobility [F (4, 40)=19.95, *P<*.001] (Figure 1b) in the FST, showing a dose-dependent manner. To exclude the possibility that GLYX-13 induced locomotor alterations in these behavioral tests, we measured the effects of GLYX-13 on locomotor activity 30 minutes before the FST. The mice treated with GLYX-13 (0.5, 5, and 10mg/kg, i.p.) or Des (10mg/kg, i.p.) did not differ from vehicle-treated mice in the number of line crossings [F (4, 40)=0.4974, *P*=.7377] (Figure 1c) or rearings [F (4, 40)=0.8229, *P*=.5184] (Figure 1c), indicating that reductions in immobility in the FST were not attributable to alterations in locomotor activity.

### GLYX-13 Administration Rapidly Reverses Depressive-Like Behaviors Induced by CUMS in Mice

This experiment investigated the effects of GLYX-13 on depressive-like behaviors induced by CUMS. Five groups of mice were used (n=9 per group):(1) No-CUMS + Vehicle, (2) CUMS + Vehicle, (3) CUMS + GLYX-13 (0.5mg/kg, i.p.), (4) CUMS + GLYX-13 (5mg/kg, i.p.), and (5) CUMS + GLYX-13 (10mg/kg, i.p.). Briefly, the mice were subjected to different stressors for 21 consecutive days. On day 22, the OFT was conducted 30 minutes after the injection of vehicle or GLYX-13. The FST was performed immediately 30 minutes after the OFT (Figure 2a). The 1-way ANOVA revealed significant differences in drug treatments in FST [F (4, 40)=11.43, *P*<.0001] (Figure 2b). Posthoc analyses indicated that exposure to CUMS in mice treated with vehicle significantly increased the immobility in the FST (*P*<.01) compared with the nonstressed mice with the same treatment. This was reversed by treatment with GLYX-13 (5 and 10mg/kg; *P*<.01) in the FST (Figure 2b). In addition, ANOVA indicated there were significant differences among all treatments in their effect on OFT behaviors:line crossings [F (4, 40) = 4.265, *P*=.0057] (Figure 2c) and rearings [F (4, 40)=9.614, *P*<.001] (Figure 2c). Posthoc tests revealed vehicle-treated CUMS mice exhibited a significant decrease in line crossings (*P*<.01) and rearings (*P*<.01), and GLYX-13 treatment (5 and 10mg/kg, i.p.) significantly reversed the decrease of line crossings (both *P<*.01) and rearings (both *P*<.01) induced by CUMS. However, 3 doses of GLYX-13 had no significant effect on line crossings (all *P* > .05) and rearings (all *P* > .05) compared with each other. Reversal of CUMS-induced behavioral despair by acute treatment confirmed the rapid-acting antidepressant potential of GLYX-13.

### Effects of GLYX-13 on Expression of pAKT, AKT, pmTOR, mTOR, peEF2, and VGF in Hippocampus of Mice

**Figure 3.**
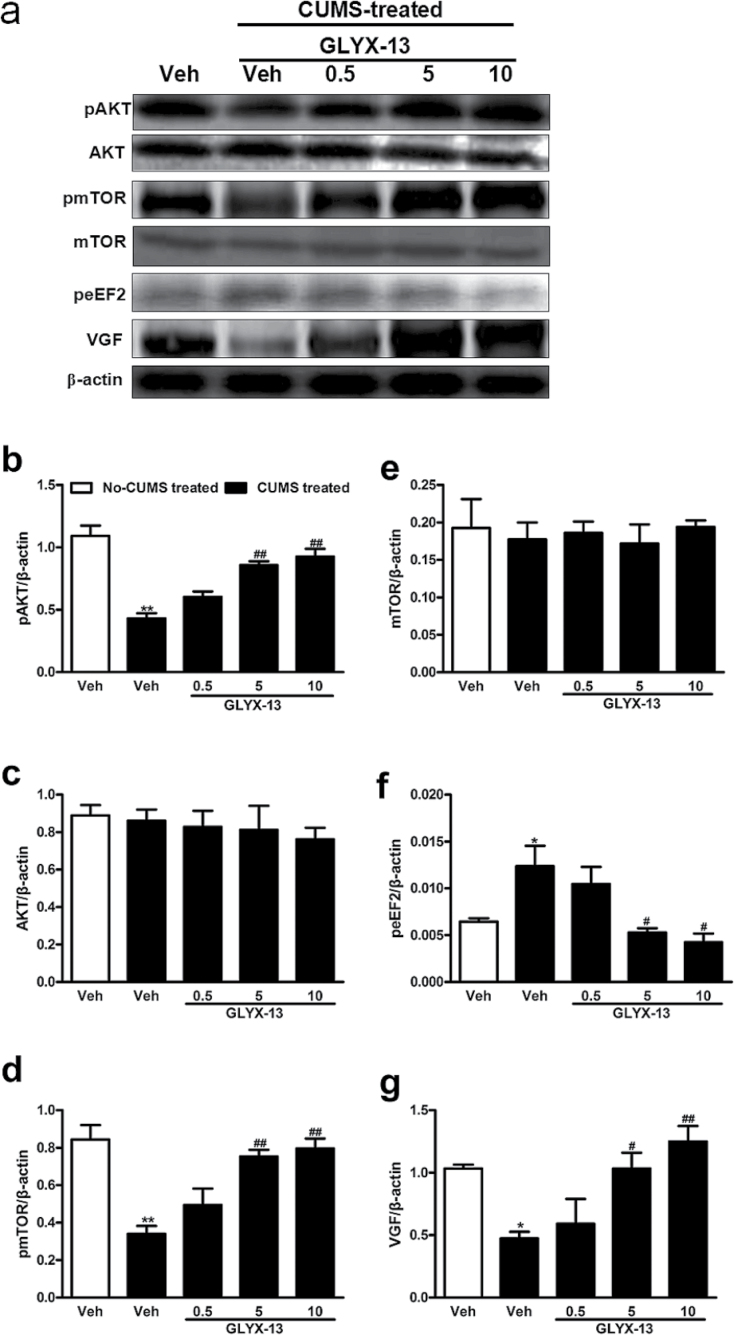
Effect of GLYX-13 on levels of pAKT, AKT, pmTOR, mTOR, peEF2. and VGF in hippocampus of mice. (a) Representative immunoblots of pAKT, AKT, pmTOR, mTOR, peEF2, and VGF detected by Western blotting with tissues from the hippocampus; the rest of the panels are quantification of the immunoblotting bands of pAKT (b), AKT (c), pmTOR (d), mTOR (e), peEF2 (f), and VGF (g). The data are expressed as mean±SEM (n=3 per group). *P<.05, **P<.01, compared with nonstressed mice treated with vehicle group; #*P<.*05, ##*P<.*01, compared with stressed mice treated with GLYX-13 group.

As shown in Figure 3, CUMS significantly decreased the expression of pAKT [F (4, 10)=22.36, *P*<.0001] (Figure 3b), pmTOR [F (4, 10)=12.13, *P*=.0007] (Figure 3d), and VGF [F (4, 10)=7.200, *P*=.0054] (Figure 3g) in hippocampus compared with the nonstressed mice treated with vehicle. However, the levels of peEF2 [F (4, 10)=6.612, *P*=.0072] (Figure 3f) was significantly increased by CUMS compared with the nonstressed mice treated with vehicle. In addition, GLYX-13 (5 and 10mg/kg, i.p.) significantly reversed the CUMS-induced changes in pAKT (*P*<.01 for both 5 and 10mg/kg in Figure 3b), pmTOR (*P*<.01 for both 5 and 10mg/kg in Figure 3d), peEF2 (*P*<.05 for both 5 and 10mg/kg in Figure 3f), and VGF (*P*<.05 for 5mg/kg and *P*<.01 for 10mg/kg in Figure 3g) in the hippocampus. In contrast, none of the treatments affected the AKT [F (4, 10)=0.3529, *P*=0.8363] (Figure 3c) and mTOR [F (4, 10)=0.1554, *P*=.9561] (Figure 3e) levels in the hippocampus of mice.

### *Vgf* Knockdown in Hippocampus Blocks the Rapid-Acting Antidepressant-Like Effects of GLYX-13 in Mice

**Figure 4.**
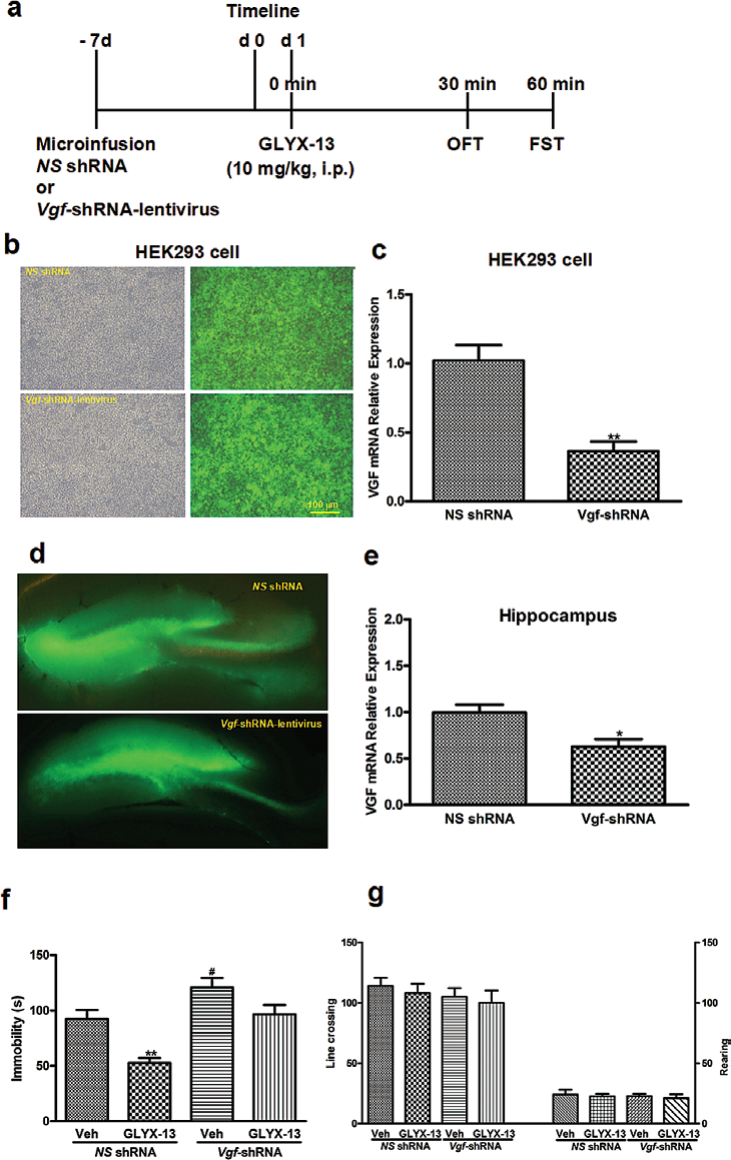
*Vgf* knockdown in hippocampus blocks the rapid-acting antidepressant-like effects of GLYX-13. (a) Experimental procedure for the test schedule. *NS* shRNA or *Vgf* shRNA were microinfused into bilateral hippocampus of mice following 7-day acclimatization. GLYX-13 (10mg/kg, i.p.) or its vehicle was administrated beginning from 7 days after the viral infusions (day 1) and then 30 or 60 minutes later, the open field test (OFT) or forced swim test (FST) was conducted, respectively. (b) *NS-*shRNA or *Vgf*-shRNA were well expressed in HEK293 cells as indicated by EGFP (green) observed under a fluorescence microscope. Scale bar=100 μm. (c) The results are expressed as 2^-△*Ct*^ [△*Ct*=*Ct* (VGF) – *Ct* (β-actin)] and normalized by the level of β-actin. (d) Microinjection sites and specific expressions of EGFP (green) in the hippocampus observed under fluorescence microscopy. Scale bars=200 μm. (e) The hippocampus tissues of 2mm in diameter around the injection site were punched out for qRT-PCR. (f) *Vgf* knockdown in the hippocampus significantly produced the depressive-like behavior and also blocked the antidepressant-like behavior in the FST of mice. (g) All the treatments had no effects on locomotor activity, reflected by the line crossings (left) and rearings (right) in mice. The data are expressed as mean±SEM (n=5 per group for VGF mRNA expression and n=9 per group for behavioral tests). **P<.01 (c), compared with *NS* shRNA group and **P<.01 (d), compared with Vehicle+*NS* shRNA group; #*P<.*05, compared with Vehicle+*NS* shRNA group.

*NS-*shRNA or *Vgf* -shRNA were microinfused into bilateral hippocampus of mice following a 7-day acclimatization. GLYX-13 (10mg/kg, i.p.) or its vehicle was administrated beginning from 7 days after the viral infusion (day 1), and then 30 or 60 minutes later, the OFT or FST was conducted, respectively (Figure 4a). Fluorescent microscopy showed that *Vgf*-shRNA and *NS-*shRNA were well expressed in HEK 293 cells, as indicated by EGFP-positive cells (green) (Figure 4b). *Vgf-*shRNA-treated cells displayed a significant decrease in VGF mRNA expression compared with *NS-*shRNA-treated cells (*P*<.01) (Figure 4c). The FST results showed that *Vgf-*shRNA microinjection plus vehicle statistically increased the duration of immobility [F (3, 32)=13.53, *P*<.0001] (Figure 4d) compared with the *NS-*shRNA + Vehicle group. In addition, GLYX-13 + Vehicle showed significantly antidepressant-like effects compared with the *NS-*shRNA + Vehicle group (*P*<.01). However, GLYX-13 treatment did not significantly alter *Vgf*-shRNA-induced depressive-like behaviors in the FST (*P* > .05). These results suggest that VGF may play an important role in GLYX-13-induced antidepressant-like effects in mice. In the OFT test, no significant alterations of line crossings [F (3, 32)=0.5336, *P*=.6626] (Figure 4e) and rearings [F (3, 32)=0.1821, *P*=.9078] (Figure 4e) were observed in mice treated with *Vgf* -shRNA or GLYX-13 alone or in combination, indicating that *Vgf*-shRNA and/or GLYX-13 did not affect locomotor activity of mice, and the antidepressant-like effects of GLYX-13 were not due to potential locomotor activity changes.

### Inhibition of VGF Activity Produced by *Vgf-*shRNA Blocks the Effects of GLYX-13 on Expression of pAKT, pmTOR, peEF2, and VGF in Hippocampus of Mice

**Figure 5.**
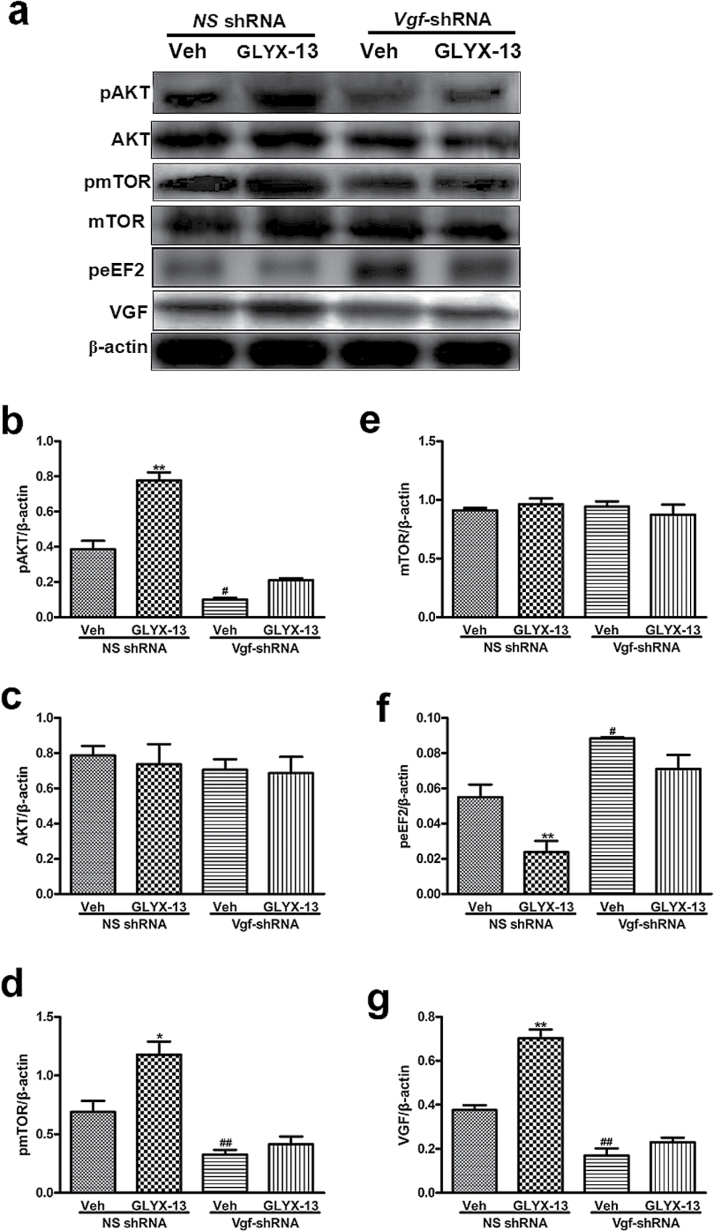
Effects of *Vgf*-shRNA and/or GLYX-13 (10mg/kg, i.p.) on the level of pAKT, AKT, pmTOR, mTOR, peEF2, and VGF in hippocampus of mice. (a) Representative immunoblots of pAKT, AKT, pmTOR, mTOR, peEF2, and VGF detected by Western blotting with tissues from the hippocampus; the rest of the panels are quantification of the immunoblotting bands of pAKT (b), AKT (c), pmTOR (d), mTOR (e), peEF2 (f), and VGF (g). The data are expressed as mean±SEM (n=3 per group). *P<.05, **P<.01, compared with Vehicle+*NS* shRNA treated group; #*P<.*05, ##*P<.*01, compared with Vehicle+*NS* shRNA treated group.

As shown in Figure 5, 2-way ANOVA revealed significant effects of GLYX-13 treatment [pAKT:F (1, 8)=54.01, *P<*.0001, Figure 5b; pmTOR:F (1, 8)=11.95, *P=*.0086, Figure 5d; peEF2:F (1, 8)=14.97, *P=*.0047, Figure 5f; VGF:F (1, 8)=43.69, *P=*.0002, Figure 5g], *Vgf-*shRNA treatment [pAKT:F (1, 8)=157.4, *P<*.0001, Figure 5b; pmTOR:F (1, 8)=41.16, *P=*.0001, Figure 5d; peEF2:F (1, 8)=41.13, *P=*.0002, Figure 5f; VGF:F (1, 8)=135.1, *P<*.0001, Figure 5g], and GLYX-13 treatment×*Vgf* -shRNA interaction [pAKT:F (1, 8)=17, *P=.*0033, Figure 5b; pmTOR:F (1, 8)=5.818, *P=.*0424, Figure 5d; peEF2:F (1, 8)=1.239, *P=.*2980, Figure 5f; VGF:F (1, 8)=20.78, *P=.*0019, Figure 5g] on the expression of pAKT, pmTOR, peEF2, and VGF in the hippocampus of mice. Posthoc analysis showed that the upregulation effects of GLYX-13 on the expression of pAKT [*P<.*01; Figure 5b], pmTOR [*P<.*05; Figure 5d], and VGF [*P<.*01; Figure 5g] were completely prevented by treatment of animals with the *Vgf*-shRNA in hippocampus. In addition, the downregulation effects of GLYX-13 on the expression of peEF2 (*P<.*01) (Figure 5f) also was completely blocked by treatment of animals with the *Vgf-*shRNA in hippocampus. By contrast, none of the treatments affected the AKT (Figure 5c) and mTOR (Figure 5e) levels in the hippocampus.

### Inhibition of PI3K Activity Blocks the Rapidly Antidepressant-Like Effects of GLYX-13 in Mice

**Figure 6.**
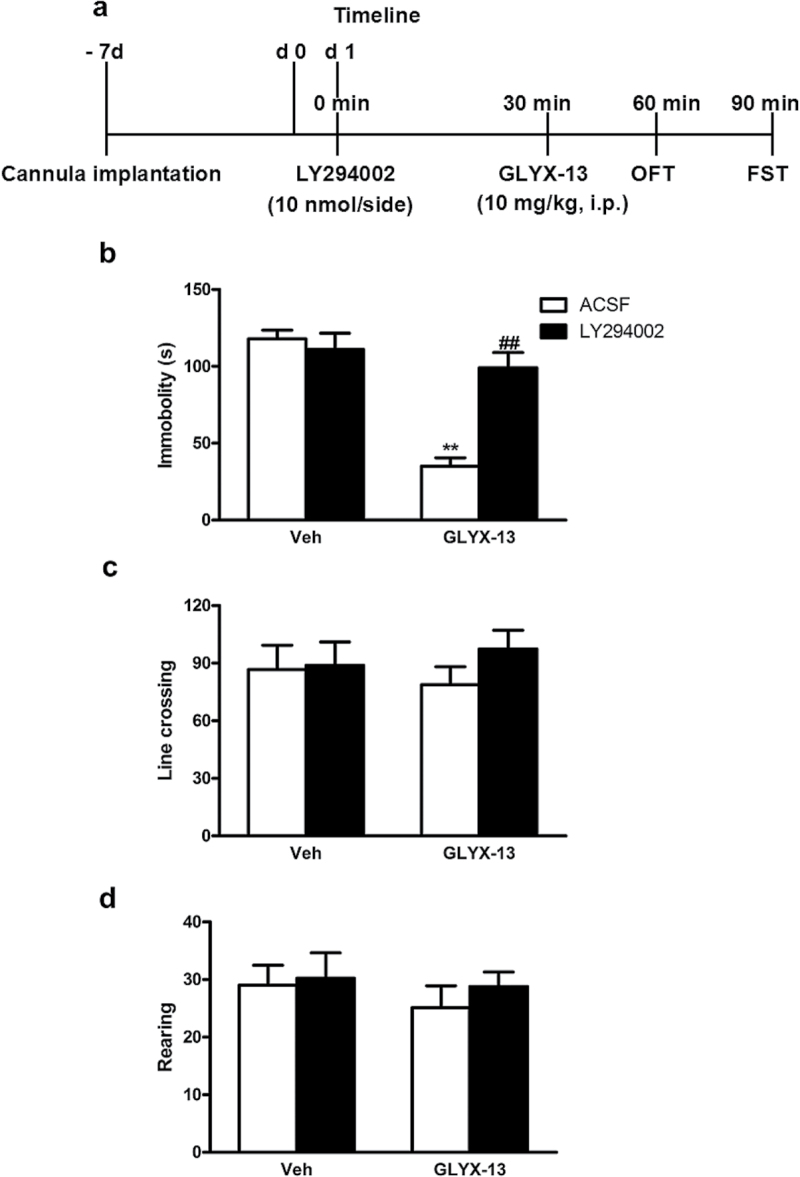
PI3K/AKT/mTOR/VGF activity mediates the antidepressant-like effects of GLYX-13 in mice. (a) Experimental procedure for the assessment of the role of PI3K/AKT/mTOR/VGF signaling in the effects of GLYX-13 (10mg/kg, i.p.). Cannula implantations were microinfused into bilateral hippocampus of mice following 7-day acclimatization. Mice were treated with LY294002 (10 nmol/side) and 30 minutes later followed by GLYX-13 (100mg/kg, i.p.) treatment. Then the open field test (OFT) was conducted 30 minutes later and the forced swim test (FST) was conducted 30 minutes after the OFT. (b) Immobility time of mice was measured. Pretreatment with LY294002 reversed the reduction of immobility time produced by GLYX-13. (c-d) All the treatments had no effects on locomotor activity, reflected by the line crossings (c) and rearings (d) in mice. The data are expressed as mean±SEM (n=9 per group). **P<.01, compared with ACSF + Vehicle group; ##*P<.*01, compared with ACSF + GLYX-13 group.

To investigate the involvement of PI3K in the antidepressant-like effect of GLYX-13, 4 groups of mice were used:(1) ACSF + Vehicle, (2) LY294002 + Vehicle, (3) ACSF + GLYX-13, and (4) LY294002 + GLYX-13. Seven days after cannula implantation, mice were pretreated with LY294002 (10 nmol/side, i.h.) or its vehicle (ACSF) 30 minutes before i.p. administration of GLYX-13 (10mg/kg) or vehicle. The OFT was carried out 30 minutes after GLYX-13 treatment, and the FST was conducted 30 minutes after the OFT (Figure 6a). As shown in Figure 6b, the effect of inhibition of PI3K produced by LY294002 in the antidepressant-like effects of GLYX-13 in the FST was examined. The 2-way ANOVA revealed significant differences for GLYX-13 treatment [F (1, 32)=32.31; *P<.*0001], LY294002 treatment [F (1, 32)=11.76; *P =*0.0017], and GLYX-13 treatment×LY294002 interaction [F (1, 32)=18.12; *P=.*0002]. Posthoc analysis showed that the antidepressant-like effect of GLYX-13 was completely prevented by treatment of animals with LY294002. Figure 6c showed that the administration of LY294002 alone or in combination with GLYX-13 was devoid of effect in the line crossings of OFT (GLYX-13 treatment [F (1, 32)=0.0006; *P=.*9802], LY294002 treatment [F (1, 32)=0.8765; *P=.*3562], and GLYX-13 treatment × LY294002 interaction [F (1, 32)=0.5416; *P=.*4671]). In addition, Figure 6d also showed that the administration of LY294002 alone or in combination with GLYX-13 did not modify the rearings of mice in the OFT (GLYX-13 treatment [F (1, 32)=0.5421; *P=.*4669], LY294002 treatment [F (1, 32)=0.4555; *P=.*5046], and GLYX-13 treatment×LY294002 interaction [F (1, 32)=0.1139; *P=.*7380]).

### Inhibition of PI3K Activity Blocks the Regulation Effects of GLYX-13 on pAKT, pmTOR, peEF2, and VGF in the Hippocampus of Mice

**Figure 7.**
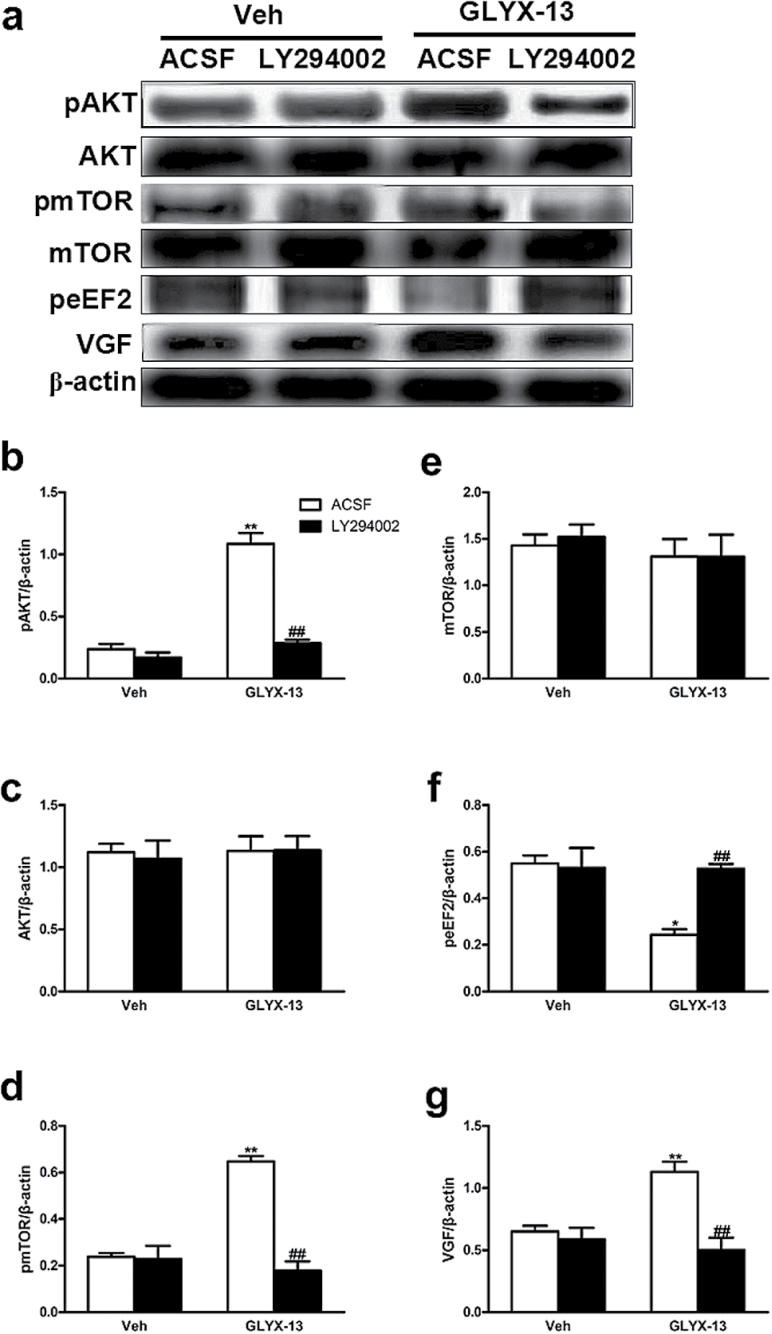
Pretreatment with LY294002 blocks the effects of GLYX-13 on expression of pAKT, AKT, pmTOR, mTOR, peEF2, and VGF in hippocampus of mice. (a) Representative immunoblots of pAKT, AKT, pmTOR, mTOR, peEF2, and VGF detected by Western blotting with tissues from the hippocampus; the rest of the panels are quantifications of the immunoblotting bands of pAKT (b), AKT (c), pmTOR (d), mTOR (e), peEF2 (f), and VGF (g). The data are expressed as mean±SEM (n=3 per group). *P<.05, **P<.01, compared with ACSF + Vehicle group; ##*P<.*01, compared with ACSF + GLYX-13 group.

As shown in Figure 7, 2-way ANOVA revealed significant effects of GLYX-13 treatment [pAKT:F (1, 8)=78.63, *P<.*0001, Figure 7b; pmTOR:F (1, 8)=37.07, *P=.*0003, Figure 7d; peEF2:F (1, 8)=10.33, *P=.*0124, Figure 7f; VGF:F (1, 8)=5.637, *P=.*0449, Figure 7g], LY294002 treatment [pAKT:F (1, 8)=63.37, *P<.*0001, Figure 7b; pmTOR:F (1, 8)=41.64, *P=.*0002, Figure 7d; peEF2:F (1, 8)=7.515, *P=.*0254, Figure 7f; VGF:F (1, 8)=17.52, *P=.*0031, Figure 7g], and GLYX-13 treatment × LY294002 interaction [pAKT:F (1, 8)=45.49, *P=.*0001, Figure 7b; pmTOR:F (1, 8)=38.65, *P=.*0003, Figure 7d; peEF2:F (1, 8)=9.734, *P=.*0142, Figure 7f; VGF:F (1, 8)=11.70, *P=.*0091, Figure 7g] on the expression of pAKT, pmTOR, peEF2, and VGF in the hippocampus of mice. Posthoc analysis showed that the upregulation effects of GLYX-13 on the expression of pAKT [*P<.*01; Figure 7b], pmTOR [*P<.*01; Figure 7d], and VGF [*P<.*01; Figure 7g] were completely prevented by treatment of animals with LY294002 in hippocampus. In addition, the downregulation effects of GLYX-13 on the expression of peEF2 (*P<.*01) (Figure 7f) also was completely blocked by treatment of animals with the LY294002 in hippocampus. By contrast, none of the treatments affected the AKT (Figure 7c) and mTOR (Figure 7e) levels in the hippocampus.

### The Antidepressant-Like Effect of GLYX-13 Is Blocked by NBQX or Rapamycin in Mice

**Figure 8.**
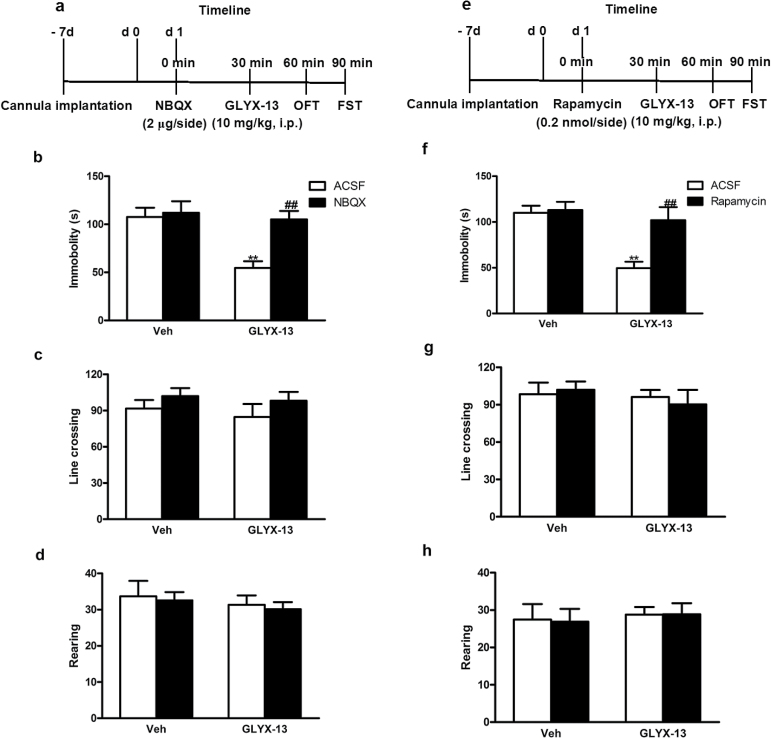
Administration of the AMPA receptor inhibitor NBQX or mTOR inhibitor rapamycin attenuates the rapid-acting antidepressant-like effectst of GLYX-13 in mice. Experimental procedure for the assessment of the role of AMPA receptor (a) or mTOR (e) in the antidepressant-like effects of GLYX-13 (10mg/kg, i.p.). Cannula implantations were microinfused into bilateral hippocampus of mice following 7-day acclimatization. Mice were treated with NBQX (2 μg/side) or rapamycin (2 nmol/side) and 30 minutes later followed by GLYX-13 (100mg/kg, i.p.). Then the open field test (OFT) was performed 30 minutes later and the FST was conducted 30 minutes after the OFT. (b, f) Immobility time of mice was measured. Pretreatment with NBQX (b) or rapamycin (f) reversed the reduction of immobility time produced by GLYX-13. All the treatments had no effects on locomotor activity, reflected by the line crossings (c, g) and rearings (d, h) in mice. The data are expressed as mean±SEM (n=9 per group). **P<.01, compared with ACSF+Vehicle group; ##*P<.*01, compared with ACSF + GLYX-13 group.

To determine whether activation of AMPA receptor or mTOR is required for the rapidly antidepressant-like effect of GLYX-13, 4 groups of mice were used in following 2 experiments respectively:(1) ACSF + Vehicle, (2) NBQX or rapamycin + Vehicle, (3) ACSF + GLYX-13, and (4) NBQX or rapamycin + GLYX-13. Seven days after cannula implantation, mice were pretreated with NBQX (2 μg/side), rapamycin (0.2 nmol/side) or their vehicle (ACSF) 30 minutes before administration of GLYX-13 (10mg/kg, i.p.) or vehicle. The OFT was carried out 30 minutes after GLYX-13 treatment, and the FST was conducted 30 minutes after the OFT (Figure 8a, e). Figure 8b showed the inhibition of the AMPA receptor by NBQX in the antidepressant-like effect of GLYX-13 (10mg/kg, i.p.) in the FST. The 2-way ANOVA revealed significant differences for GLYX-13 treatment [F (1, 32)=9.901; *P=.*0036], NBQX treatment [F (1, 32)=8.222; *P=.*0073], and GLYX-13 treatment×NBQX interaction [F (1, 32)=5.770; *P=.*0223]. Posthoc analysis showed that the antidepressant-like effect of GLYX-13 was completely prevented by treatment of animals with the NBQX. Figure 8c showed that the administration of NBQX alone or in combination with GLYX-13 was devoid of effect in the line crossings of OFT (GLYX-13 treatment [F (1, 32)=0.446; *P=.*5087], NBQX treatment [F (1, 32)=2.130; *P=.*1542], GLYX-13 treatment×NBQX interaction [F (1, 32)=0.03646; *P=.*8498]). In addition, Figure 8d also showed that the administration of NBQX alone or in combination with GLYX-13 did not modify the rearings of mice in the OFT (GLYX-13 treatment [F (1, 32)=0.6673; *P=.*4200], NBQX treatment [F (1, 32)=0.1591; *P=.*6926], GLYX-13 treatment×NBQX interaction [F (1, 32)=0.0004; *P=.*9850]). We further determined whether the requirement for activation of mTOR signaling in the rapid actions of GLYX-13. Figure 8f showed the inhibition of mTOR by rapamycin in the antidepressant-like effect of GLYX-13 (10mg/kg, i.p.) in the FST. The 2-way ANOVA revealed significant differences for GLYX-13 treatment [F (1, 32)=12.85; *P<.*0011], rapamycin treatment [F (1, 32)=7.682; *P =* .0092], and GLYX-13 treatment × rapamycin interaction [F (1, 32)=6.107; *P=.*0190]. Posthoc analysis showed that the antidepressant-like effect of GLYX-13 was completely prevented by treatment of animals with rapamycin. Figure 8g showed that the administration of rapamycin alone or in combination with GLYX-13 was devoid of effect in the line crossings of OFT (GLYX-13 treatment [F (1, 32) =0.6618; *P=.*4219], rapamycin treatment [F (1, 32)=0.0181; *P* *=*.8938], GLYX-13 treatment×rapamycin interaction [F (1, 32)=0.2964; *P* *=*.5899]). In addition, Figure 8h also showed that the administration of rapamycin alone or in combination with GLYX-13 did not modify the rearings of mice in the OFT (GLYX-13 treatment [F (1, 32)=0.2648; *P=.*6104], rapamycin treatment [F (1, 32)=0.0047; *P=.*9457], and GLYX-13 treatment×rapamycin interaction [F (1, 32)=0.0106; *P=.*9187]).

## Discussion

Interestingly, our present study provided the first evidence that the rapid-acting antidepressant-like effect of GLYX-13 in the FST involves PI3K activation, increased mTOR signaling, and VGF activation. In addition, our results further demonstrated that the GLYX-13-induced antidepressant-like effects required AMPA/kainate receptor and mTOR activation, as evidenced by the ability of NBQX and rapamycin to abolish the antidepressant-like effect of GLYX-13, respectively. Moreover, the fast-acting antidepressant effect elicited by GLYX-13 is associated with VGF in hippocampus of mice.

Increasing evidence has shown that the well-documented delay in antidepressant efficacy (Adell et al., 2005) may be attributable to neural adaptive mechanisms to reverse the damage of stress in the hippocampus, including changes in neurotrophic factors (Castren, 2005; Dranovsky and Hen, 2006; Warner-Schmidt and Duman, 2006). The neurotrophic hypothesis of depression is based on the stress-induced downregulation and rapid-acting antidepressant-induced upregulation of BDNF and neuropeptide VGF (nonacronymic) expression in the brain (Adlard and Cotman, 2004; Thakker-Varia et al., 2010; Nasca et al., 2013; Lin et al., 2014). VGF is upregulated by both BDNF and 5-HT treatment, and VGF protein in the hippocampus is reduced in animals subjected to behavioral models of depression (Thakker-Varia et al., 2007). This hypothesis is supported by previous research indicating that the administration VGF-derived peptide TLQP-62 into the brain produced sustained and rapidly antidepressant-like effects (Lin et al., 2014). Additionally, growing studies suggest that the rapid-acting antidepressant-like effects of ketamine are mediated by molecular alterations to the mTOR, a serine/threonine kinase and key component of the insulin-signaling pathway and then reduces the expression of eEF2, which prevents protein translation of BDNF (Li et al., 2010; Autry et al., 2011; Monteggia et al., 2013). Furthermore, BDNF regulates neuronal mTOR function via AKT and PI3K, creating a positive feedback loop of BDNF production following the activation of mTOR by ketamine (Hay and Sonenberg, 2004; Hoeffer and Klann, 2010), suggesting that the production of BDNF occurs rapidly and may underlie the fast behavioral response to ketamine. However, to date, whether neuropeptide VGF also plays an important role in the rapid onset antidepressant-like effects of antidepressants remains unknown. In addition, given that the GLYX-13 is a recently developed tetrapeptide (Thr-Pro-Pro-Thr) that acts as a glycine-site modulator at the NMDAR (Moskal et al, 2005) with therapeutic potential as a cognitive enhancer (Moskal et al., 2005) and antidepressant (Moskal et al., 2014), whether the PI3K/AKT/mTOR/VGF signaling also involve in the rapid-acting antidepressant-like effects of GLYX-13 is not clear. In support of this hypothesis, we confirmed that GLYX-13, a novel NMDAR glycine-site functional partial agonist, produces an antidepressant-like effect in the FST of mice. Our present results are consistent with previous studies that revealed that GLYX-13 produced rapid-acting antidepressant-like effects in the rat Porsolt test and novelty-induced hypophagia test (Burgdorf et al., 2013). The previous studies demonstrated that the following reasons may explain the rapid-acting antidepressant-like effects of GLYX-13 in the current work. First, GLYX-13 has been shown to readily cross the blood–brain barrier, showing a brain uptake index of 80% (Moskal et al, 2005). Second, it enhances the magnitude of long-term potentiation of synaptic transmission while reducing long-term depression (Zhang et al., 2008). Third, GLYX-13 has been shown to have a critical role in the induction of metaplasticity (Yang et al, 2011). Our current data also showed that CUMS for 3 weeks caused a reduction of the pAKT, pmTOR, and VGF expression and a increase of peEF2 in hippocampus of mice and that these stress-induced changes were prevented by acute GLYX-13 treatment. To the best of our knowledge, this study is the first to provide evidence for the increased PI3K/AKT/mTOR/VGF signaling following acute treatment with GLYX-13. Thus, the corrective action of GLYX-13 on brain PI3K/AKT/mTOR/peEF2 signaling-mediated VGF levels may be in line with its rapid-acting antidepressant activity in mice.

Further, VGF shRNA-mediated *Vgf* knockdown in hippocampus has been used to investigate mechanisms underlying the acute behavioral actions of GLYX-13. Downregulation of VGF in hippocampus produced depressive-like behaviors, as indicated by increased immobility in the FST. Furthermore, we compared the antidepressant-like effect of GLYX-13 with that of combined GLYX-13 and *Vgf* shRNA. If VGF was important, inhibition of the VGF in hippocampus should block the rapid-acting antidepressant-like effects of GLYX-13. Indeed, the downregulation of VGF in hippocampus significantly increased the immobility in the FST compared with the *NS* shRNA-treated group and also blocked the antidepressant-like effects of GLYX-13 when combined with GLYX-13, supporting the important role of VGF in the mediation of rapid-acting antidepressant activity of GLYX-13. Furthermore, our present study also demonstrated that *Vgf* knockdown in hippocampus decreased the expressions of pAKT, pmTOR, and VGF and increased the levels of peEF2. The following reasons may explain the relationship between PI3K/AKT/mTOR and VGF in our present work. The VGF may act downstream of BDNF and exert rapid-acting antidepressant-like effects through TrkB receptor (Alder et al., 2003; Lin et al., 2014) in hippocampus. In addition, BDNF-induced TrkB activation stimulates the PI3K/AKT signaling pathways, which play a principal role in promoting neuronal survival and synaptic plasticity and antidepressant-like actions (Numakawa et al., 2010, 2013), suggesting that VGF may feedback regulate the PI3K/AKT/mTOR signaling in hippocampus of mice. Our results not only are consistent with rapid-acting antidepressant-like effects of GLYX-13 (Burgdorf et al., 2013; Moskal et al., 2014) and VGF (Lin et al., 2014) but also extended the findings for the first time to the role of VGF in the rapid-acting antidepressant-like effects of GLYX-13 and in the regulation of PI3K/AKT/mTOR signaling.

As the downstream regulatory signaling pathway of GLYX-13 in our current work, PI3Ks are a large family of intracellular signal transducers. The role of the PI3K pathway has been implicated in the regulation of cell growth, survival, proliferation, and movement (Astle et al., 2011; Nedachi et al., 2011). Numerous studies have also implicated PI3K in depression and anxiety (Leibrock et al., 2013; Moretti et al., 2014). Another interesting result of our present study showed that inhibition of AKT, following blockade of PI3K by LY294002, prevented GLYX-13–produced antidepressant-like effects and upregulation on PI3K/AKT/mTOR/VGF signaling. These results not only indicate that blocking PI3K is most likely involved in the fast-acting effects of GLYX-13 but also confirms that rapid increases in VGF produced by activation of PI3K/AKT/mTOR signaling are useful markers of novel rapid-acting antidepressants. One limitation of the current study is that several reports have shown that LY294002 is not exclusively selective for the PI3Ks and could in fact act on other lipid kinases and additional apparently unrelated proteins (Gharbi et al., 2007). Therefore, the involvement of other tyrosine kinases in the antidepressant-like effects of GLYX-13 cannot be excluded. Future studies should be aimed at using the selective PI3K inhibitor to understand of PI3K/AKT signaling in the modulation of depressive-like behavior and particularly in relation to the rapid-acting antidepressant-like effects of GLYX-13. Additionally, it has recently been reported that the antidepressant-like effects of GLYX-13 were prevented by preadministration of NBQX (Burgdorf et al., 2013). The requirement for AMPA receptor is consistent with our current finding that NBQX also blocked the rapid-acting antidepressant-like effects of GLYX-13. Further, our novel finding that mTOR is involved in the VGF upregulation contributes to the antidepressant-like effects of GLYX-13. Our results showed that pretreatment with rapamycin completely abolished the effects of GLYX-13.

## Conclusion

To our knowledge, our present study is the first to report that GLYX-13 activate the PI3K/AKT/mTOR signaling pathway, leading to VGF activation in hippocampus of mice. Taken together, alterations in the expression of the proteins investigated in the present study may contribute to the molecular basis of depressive-like behaviors and changes in brain of mice and could provide novel insights into the development of new therapeutic approaches having greater efficacy against major depression.

## Statement of Interest

None.
